# A biomechanical comparison between robotic and conventional total knee arthroplasty (TKA) in resection accuracy: a meta-analysis on cadaveric specimens

**DOI:** 10.1186/s40634-023-00587-y

**Published:** 2023-03-30

**Authors:** Sean B. Sequeira, Grant T. Duvall, Henry Boucher

**Affiliations:** Department of Orthopaedic Surgery, MedStar Union Memorial Hospital, 3333 North Calvert, Street, Suite 400, Baltimore, MD 21218 USA

**Keywords:** Total knee arthroplasty, Biomechanics, Cadaveric, Resection error, Sagittal, Coronal

## Abstract

**Purpose:**

Robotic total knee arthroplasty (TKA) has seen a rapid increase in utilization with recent literature suggesting that implant accuracy and resection are better optimized than in conventional TKA. The purpose of this study was to evaluate the biomechanical properties of robotic-assisted versus conventional TKA in minimizing biplanar femoral and tibial resection error in cadaveric specimens.

**Methods:**

A systematic review and meta-analysis was performed by searching through PubMed, Cochrane library, and Embase to identify studies that analyzed the biomechanical properties of robotic assisted and conventional TKA, according to standard PRISMA guidelines. Evaluated outcomes included femoral coronal resection error (deg), femoral sagittal resection error (deg), tibial coronal resection error (deg), and tibial sagittal resection error (deg).

**Results:**

Seven studies met inclusion criteria, including a total of 140 cadaveric specimens (robotic: 70, conventional: 70), for resection accuracy between robotic and conventional TKA. Pooled analysis from seven studies revealed a significant difference in femoral coronal and sagittal resection error in favor of robotic systems compared to conventional systems (*p* < 0.001 & *p* < 0.001, respectively). The pooled analysis from seven studies revealed a significant difference in tibial sagittal resection error in favor of robotic systems compared to conventional systems following TKA (*p* = 0.012). *Posthoc* power analysis revealed a power of 87.2%.

**Conclusion:**

The use of robotic TKA is associated with lower femoral coronal, lower femoral sagittal and tibial sagittal resection error compared to conventional TKA. It should be noted that these findings are purely biomechanical – surgeons should interpret these findings along with clinical differences between conventional and robotic systems to determine which system is best for each patient.

## Background

Total knee arthroplasty (TKA) is one of the most successful surgeries performed today and, as the surgery and patient population continues to be optimized, the rate at which TKAs are being performed is exponentially increasing [6]. Though the evidence suggesting that a low resection error improves patient reported outcomes and satisfaction is not particularly compelling, low resection error has been associated with a reduced risk of revision [16]. Initially, the use of computers for navigation and patient-specific instrumentation had been introduced to knee arthroplasty to enhance resection accuracy, though, most recently, robotics has been implemented to further refine the accuracy of the key conventional femoral and tibial cuts of TKA.

Robotic systems are equipped with several enhancements to control and optimize the resection accuracy during a TKA. Robotic systems generally confine the saw blade to a single anatomic plane to enhance the precision of the cut or confine the blade to the preoperative surgical plan [14]. Other systems position guides relative to the bone or use burring tools during the milling process of the resected surface, all of which theoretically optimizes the resection accuracy. A large meta-analysis of clinical studies revealed that robotic systems achieve higher knee society scores (KSS) and HSS (Hospital for Special Surgery Score) scores compared to conventional TKA [10]. Furthermore, in a retrospective review of 351 patients, robotic TKA demonstrated better and more consistent radiographic outcomes compared to conventional TKA at a mean of 11 years of follow-up [4].

Though there are several biomechanical studies that compare the resection accuracy of robotic versus conventional systems for cuts during TKA, there is no consensus on the biomechanical superiority between the two systems for femoral and tibial coronal and sagittal cuts. Therefore, the objective of this study was to systematically review the literature and analyze the biomechanical properties of robotic versus conventional systems for resection accuracy. We hypothesized that robotic systems would exhibit more accurate cuts, resulting in less error in femoral and tibial coronal and sagittal planes.

## Methods

This review is registered with PROSPERO, registration CRD42022314787. The standard Preferred Reporting Items for Systematic Reviews and Meta-Analyses (PRISMA) guidelines were utilized to conduct this investigation. Three databases, including Embase, Cochrane Library, and Pubmed, were searched by two reviewers up to June 1^st^, 2022 using the search string ‘conventional’ AND ‘robotic’ AND ‘cadaveric.’ Studies were excluded if they predominantly evaluated resection error *in* vivo, studies that did not evaluate resection error as a primary outcome, clinical studies, and studies without full-text available. Data extraction from each study was performed independently and reconciled by a second author. There was no need for funding or a third party to obtain any collected data.

In order to evaluate bias within the study, a validated scale, The Quality Appraisal for Cadaveric Studies (QUACS), was implemented. The QUACS scale comprises thirteen items each of which is assigned a 0, for absent, or 1, for present, within the manuscript. A score above 75% was considered satisfactory and met inclusion within the study.

The outcomes that were evaluated within this meta-analysis were femoral coronal error (deg), femoral sagittal error (deg), tibial coronal error (deg), and tibial sagittal error (deg). In the seven studies, these outcomes were reported as the degrees of error from the planned/ideal resection. Of the seven studies that evaluated resection accuracy, all seven commented on femoral and tibial coronal and sagittal error, between robotic and conventional systems.

When standard deviations were absent and only standard errors were reported, standard deviations were computed utilizing the methodology described in the Cochrane Handbook for Systematic Reviews of Interventions (version 6.2.0). Weighted averages were calculated for all quantitative outcomes which were ultimately categorized into a Forest plot when data from two or more studies were available. Using a random-effects model, standardized mean differences (SMD) with 95% confidence intervals (CI) were calculated and embedded within the forest plot. A random-effects model was used in order to incorporate the heterogeneity between each included study into the final statistical analysis. In order to quantify the degree of heterogeneity due to between-study characteristics, *I*^*2*^ statistics were used to calculate heterogeneity. Meta-analyses statistics and generation of forest plots figures were performed using OpenMetaAnalyst, which implements metafor R console code.

## Results

**Fig. 1:**
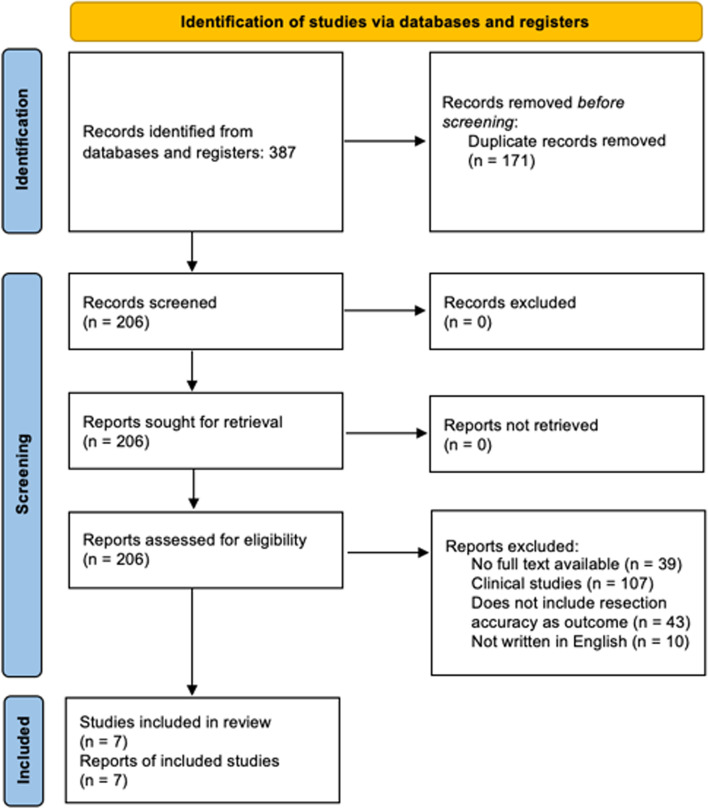
PRISMA flowchart of included studies

A total of 206 studies were reviewed by title and/or abstract to determine study eligibility based on aforementioned inclusion criteria (Fig. 1). Seven studies, including a total of 140 cadaveric specimens, met inclusion criteria for robotic versus conventional TKA [4, 5, 7, 9, 11–13]. These studies are summarized in Tables 1 and 2.

**Table 1:** Robotic and conventional surgery techniques of included studies

Study	Techniques Compared	Conventional System	Robotic System	Resection Verification	Implant System
Singh et al	Robotic; conventional	ATTUNE Intuition	VELYS Robotic System (Depuy)	CT scan; White-light scan (Artec Spacer Spider 3D Scanner)	ATTUNE cruciate-retaining (DePuy)
Doan et al	Robotic; conventional	ATTUNE Intuition	VELYS Robotic System (Depuy)	Optical Scan (Artec Spacer Spider 3D Scanner)	ATTUNE cruciate-retaining (Depuy)
Seidenstein et al	Robotic; conventional	Persona (2), NextGen (1), Vanguard (1)	ROSA Knee System (Zimmer Biomet)	Optical navigation (Sesamoid Plasty V2)	Persona (2), NextGen(1), Vanguard (1)
Kim et al	Robotic; conventional	Zimmer NextGen	ORTHODOC (Curexo Inc, South Korea)	3D Ct Scan (BrightSpeed Edge WCT 8-channel scanner)	NextGen CR system
Hampp et al	Robotic; conventional	Stryker CR manual system	Mako System (Stryker)	Optical tracking navigation device	Stryker cruciate retaining instrumentation
Moon et al	Robotic; conventional	Zimmer NextGen system jigs	ROBODOC (Curexo Inc, South Korea)	3D CT Scan (BrightSpeed Edge WCT eight-channel scanner)	NextGen (Zimmer, IN)
Scholl et al	Robotic; conventional	Stryker Triathlon system	Stryker Mako system	CT Scan	Stryker

**Table 2:** Available outcomes within included studies

Study	N (Robotic; conventional)	Cadaver Age	Femoral Coronal Resection error (deg)	Femoral Sagittal Resection error (deg)	Tibial Coronal Resection error (deg)	Tibial Sagittal Resection error (deg)
Singh et al	40 – 20;20	70.4 ± 8.2 years	+	+	+	+
Doan et al	40 – 20;20	70.4 ± 8.2 years	+	+	+	+
Seidenstein et al	20 – 10;10	77 [51–94] years	+	+	+	+
Kim et al	20 – 10;10	N/A	+	+	+	+
Hampp et al	12 – 6;6	74 (53–93) years	+	+	+	+
Moon et al	20 – 10;10	N/A	+	+	+	+
Scholl et al	12 – 6;6	79 (68–86) years	+	+	+	+

### Conventional TKA surgical technique

Six studies utilized a median parapatellar approach with one study implementing a mini-medial parapatellar approach in standard fashion [7]. Knee arthroplasty was performed in standard fashion with conventional system jigs according to the surgeon’s preference (Table 1).

### Robotic TKA surgical technique

All robotic systems among included studies used a preoperative plan with a 3D model modeled for the cadaver’s anatomy prior to incision. A standard medial parapatellar approach was utilized in six studies, with one study utilizing a mini-medial parapatellar approach to visualize the articular surface. Tracker arrays were fixed to the femur and tibia. The limb alignment and surface anatomy were registered. The robotic arm was then used to perform the cuts with either saw or burr, or position cutting guides that allowed for manual saw use. All robotic systems included real-time tracking during the cuts to account for bony or leg movement intraoperatively. In the study by Kim et al. a proprietary cutting and tissue-sparing tunneling device was utilized which accommodated for the minimally invasive approach used [7]. The ROBODOC and ORTHODOC systems, utilized in the Kim et al. and Moon et al. studies respectively, are fully active robotic systems which do not provide intraoperative tactile feedback in the way semi-active robotic systems do [7, 9]. The MAKO system, utilized in Hampp et al. and Scholl et al.’s studies, utilizes haptic technology based upon preoperative CT scan to identify bony landmarks for resection [5, 11]. The ROSA system, utilized in Seidenstein et al.’s study, uses a cutting guide attached to the robotic arm with either radiographs or image-free analysis to define bony landmarks for resection. The VELYS system, utilized in the Doan et al. and Singh et al. studies, does not utilize preoperative imaging with bone-mounted tibial and femoral arrays to identify anatomic landmarks and a robotic arm with a saw blade attached to perform resection.

The risk of bias and methodologic quality of the included studies were assessed using the QUACS scale, which has been previously validated [15]. The mean QUACS score was 85.7% (range 76.9% – 92.3%). All seven studies satisfied the threshold for a satisfactory methodologic quality (> 75%) (Table 3).

**Table 3:** QUACS scores for included studies

Study	QUACS Score
Doan et al	92.3%
Singh et al	76.9%
Seidenstein et al	92.3%
Kim et al	76.9%
Hampp et al	92.3%
Moon et al	84.6%
Scholl et al	84.6%

### Femoral coronal resection error

**Fig. 2:**
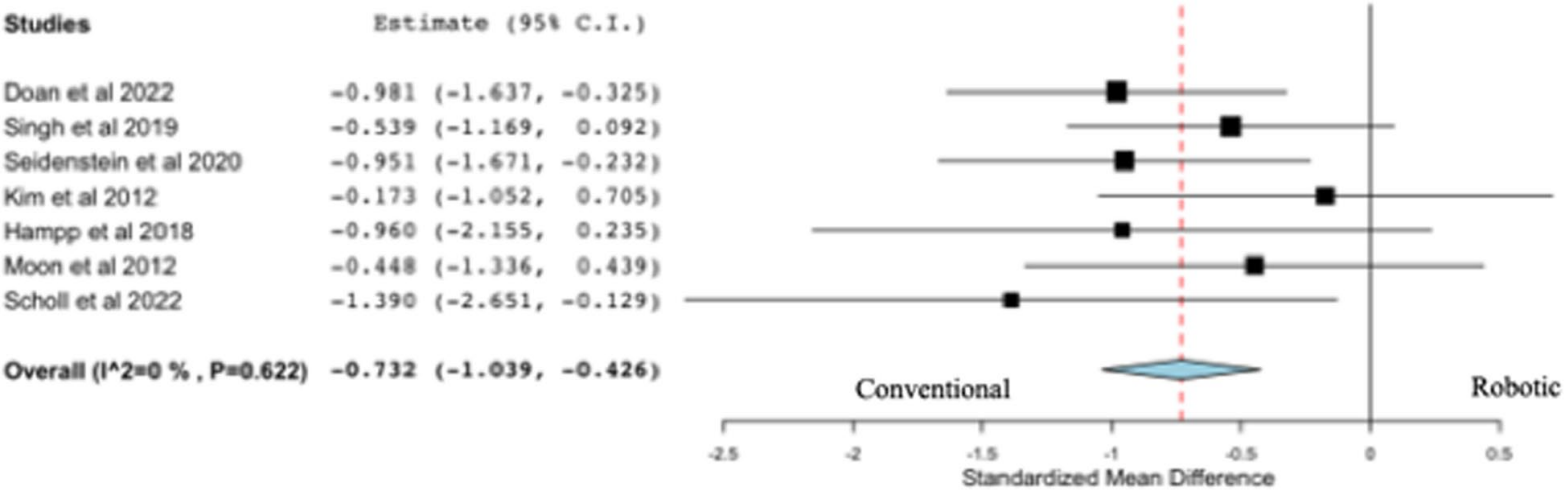
Forest plot of standardized mean difference in femoral coronal resection error between robotic and conventional TKA, favoring robotic TKA

All seven studies on resection accuracy reported on femoral coronal resection error (deg), comparing robotic versus conventional systems (Fig. 2). Four out of seven studies concluded that the use of robotic systems for TKA was associated with a significantly lower femoral coronal resection error than conventional systems, while the remaining studies concluded there was no difference between robotics and conventional systems following TKA. The pooled analysis from seven studies revealed a statistically significant difference in favor of robotic systems compared to conventional systems following TKA (*p* < 0.001). *Posthoc* power analysis revealed seven studies of 138 cadaveric specimens achieved a power of 87.2%.

### Femoral sagittal resection error

**Fig. 3:**
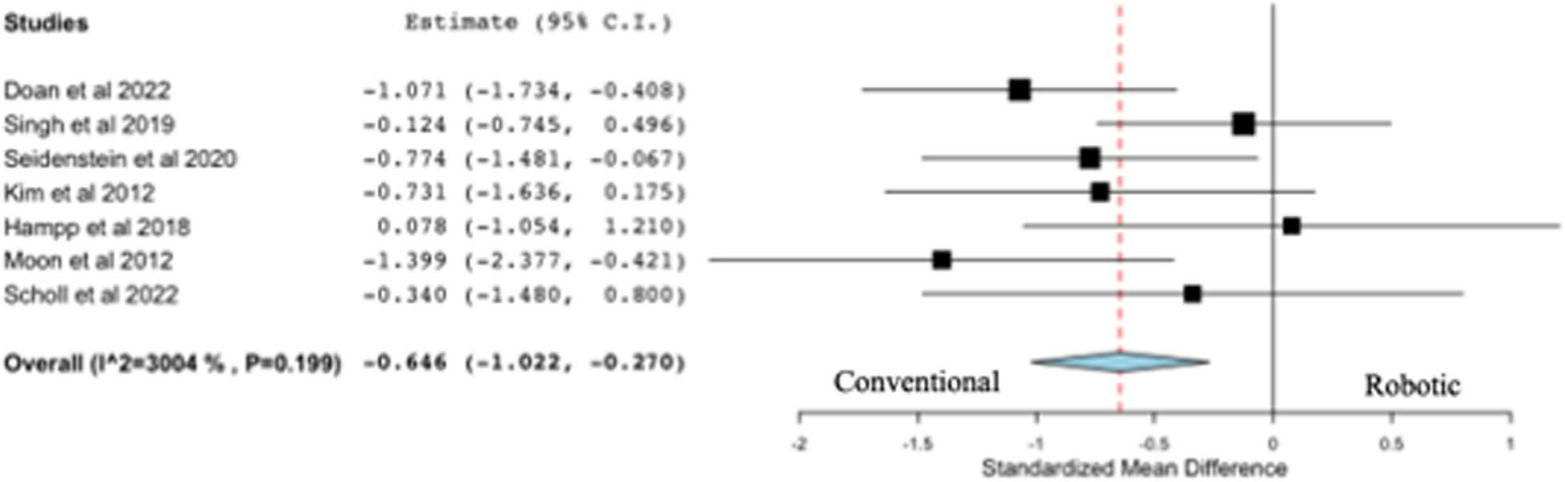
Forest plot of standardized mean difference in femoral sagittal resection error between robotic and conventional TKA, favoring robotic TKA

All seven studies on resection accuracy reported on femoral sagittal resection error (deg), comparing robotic versus conventional systems (Fig. 3). Three out of seven studies concluded that the use of robotic systems for TKA was associated with a significantly lower femoral sagittal resection error than conventional systems, while the remaining studies concluded there was no difference between robotics and conventional systems following TKA. The pooled analysis from seven studies revealed a statistically significant difference in favor of robotic systems compared to conventional systems following TKA (*p* < 0.001).

### Tibial coronal resection error

**Fig. 4:**
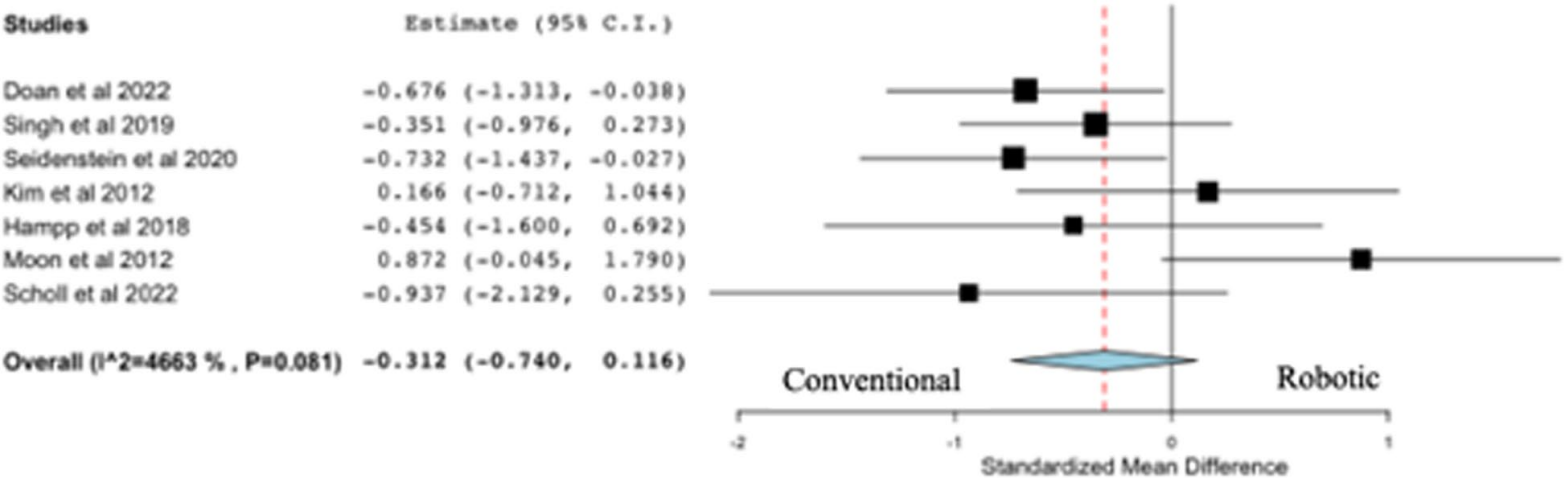
Forest plot of standardized mean difference in tibial coronal resection error between robotic and conventional TKA, favoring robotic TKA

All seven studies on resection accuracy reported on tibial coronal resection error (deg), comparing robotic versus conventional systems (Fig. 4). Four out of seven studies concluded that the use of robotic systems for TKA was associated with a significantly lower tibial coronal resection error than conventional systems, while the remaining studies concluded there was no difference between robotics and conventional systems following TKA. The pooled analysis from seven studies failed to reveal a statistically significant difference in favor of robotic systems compared to conventional systems following TKA (*p* = 0.153).

### Tibial sagittal resection error

**Fig. 5:**
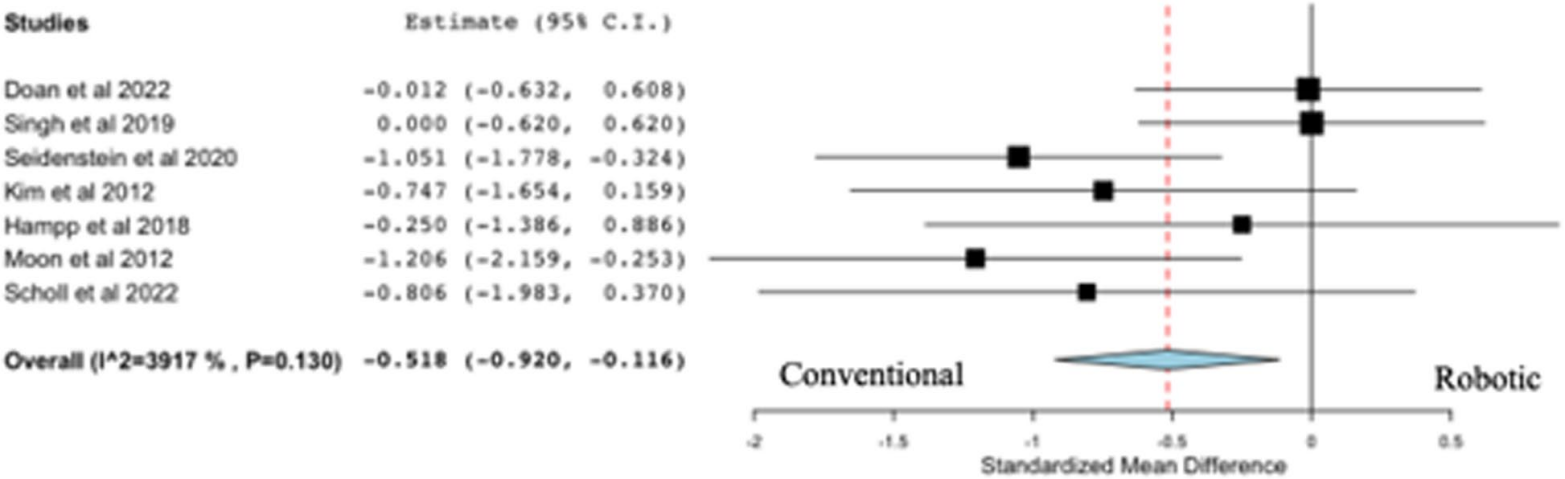
Forest plot of standardized mean difference in tibial sagittal resection error between robotic and conventional TKA, favoring robotic TKA

All seven studies on resection accuracy reported on tibial sagittal resection error (deg), comparing robotic versus conventional systems (Fig. 5). Two out of seven studies concluded that the use of robotic systems for TKA was associated with a significantly lower tibial sagittal resection error than conventional systems, while the remaining studies concluded there was no difference between robotics and conventional systems following TKA. The pooled analysis from seven studies revealed a statistically significant difference in favor of robotic systems compared to conventional systems following TKA (*p* = 0.012).

## Discussion

Robotic systems for TKA have been heavily implemented recently, owing to the theoretical benefit of improved resection accuracy over conventional systems during TKA. In this meta-analysis, our findings suggest that robotic-guided systems are associated with a lower resection error in the femoral coronal plane, femoral sagittal plane, and tibial sagittal planes. Interestingly, robotic-guided systems exhibited no difference in resection error in the tibial coronal plane compared to conventional systems.

The way in which robotic assisted TKA systems theoretically achieve higher accuracy than conventional systems depends upon the blueprint of the robotic system itself. Some systems constrain the blade to a single anatomic plane when cuts are being performed rather than the freehand capabilities of conventional systems. Moreover, these systems confine cuts to the preoperative surgical plan, theoretically disallowing for variability [6]. Other robotic systems help to enhance resection accuracy by positioning resection guides relative to bone and/or implementing burring tools during milling of the resection surface [2, 3]. Our findings suggest that robotic systems do improve resection accuracy in the femoral coronal, femoral sagittal, and tibial sagittal planes which is likely associated with the aforementioned constraints the robotic system places on the major femoral and tibial cuts.

Despite the robotic system demonstrating lower resection error in the femoral coronal, femoral sagittal, and tibial sagittal planes, there was no difference between robotic and conventional systems in the tibial coronal plane, though it did trend toward significance. This is an interesting finding in the presence of differences between robotic and conventional systems in the sagittal plane of the tibia and both femoral planes. These findings may suggest that although the robotic system is accurate at producing a neutral proximal tibial cut in the coronal plane, the surgeons among the included studies are skilled enough to, even in a conventional system, achieve a similarly neutral proximal tibial resection, thereby yielding an insignificant result between robotic and conventional systems. Indeed, the absolute value of both the conventional and robotic tibial coronal resection error were, comparatively, the lowest out of the four resection errors evaluated in this meta-analysis. However, future studies would need to more comprehensively evaluate this phenomenon to validate it. Nevertheless, studies on the clinical value of tibial component coronal alignment suggest that a more accurate proximal tibial resection that yields a better tibial coronal resection error is not associated with higher range of motion, KSS clinical, or KSS functional scores [8].

Clinical studies suggest that robotic TKA exhibit patient reported outcomes and fewer postoperative complication compared to conventional TKA, likely associated with enhanced resection accuracy as revealed in our findings. In a systematic review that evaluated the Stryker MAKO robotic system, 36 studies demonstrated that the CT-based robotic system reduces postoperative pain and improves implant positioning compared to conventional TKA systems [1]. Onggo and colleagues performed a meta-analysis on robotic knee arthroplasty and concluded that though robotic TKA was associated with lower blood loss and superior alignment in different axes, the authors questioned if the difference between robotics and conventional systems in these metrics was clinically meaningful [10]. Another meta-analysis performed by Agarwal et al. revealed that patients who underwent robotic TKA achieved higher Hospital for Special Surgery (HSS) and other patient-reported outcome scores compared to the conventional cohort, suggesting that robotic systems can reliably achieve stronger clinical outcomes than conventional systems [2]. Importantly, despite these better clinical outcomes, future studies must be performed to clinically correlate the differences in resection accuracy revealed in our findings to determine at what point a difference in resection accuracy impacts clinical and patient reported outcomes.

Though this meta-analysis is the first of its kind to systematically review the existing biomechanics literature on resection accuracy between robotic and conventional systems for TKA, it does have limitations. Heterogeneity in the robotic and conventional techniques used to perform the TKA limits the conclusions that can be reasonably drawn with this investigation, which is evident by the I^2^ statistic for all biomechanical outcome variables. Nevertheless, there are several similarities amongst these studies that make this review and meta-analysis an important anchor for comparison between robotic and conventional systems. Furthermore, this review only included seven biomechanical studies which may suggest that the conclusions drawn within this study are not adequately powered – however, when considering the number of cadaveric specimens and power analyses within included studies, it is reasonable to infer that this meta-analysis is equivalently powered.

## Conclusion

The use of robotic TKA is associated with lower femoral coronal, lower femoral sagittal and tibial sagittal resection error compared to conventional TKA. It should be noted that these findings are purely biomechanical – surgeons should interpret these findings along with clinical differences between conventional and robotic systems to determine which system is best for each patient.

## Data Availability

The datasets used and/or analyzed during the current study are available from the corresponding author on reasonable request. Furthermore, all data used in this study was publicly available within each study included within this meta-analysis.
